# Parent-Child Disconnectedness and Mental Health in Older Europeans: Regional Differences in SHARE

**DOI:** 10.1093/geroni/igaf122.1315

**Published:** 2025-12-31

**Authors:** Deborah Carr, Lisa Jessee

**Affiliations:** Boston University, Boston, Massachusetts, United States; University of Cologne, Cologne, Nordrhein-Westfalen, Germany

## Abstract

Parent-child disconnectedness – or the lack of contact to at least one child – can negatively affect older adults’ mental health. However, studies have largely overlooked how the psychological impact varies across cultural contexts. In regions with a strong cultural emphasis on family ties, like Southern Europe, the psychological impact of disconnectedness may be more severe, while in societies with more individualistic cultural norms, the consequences could be less pronounced. To address this gap, we used pooled data from eight waves (1, 2, 4, 5, 6, 7, 8, 9) of the Survey of Health, Ageing and Retirement in Europe (SHARE) and compared the association of parent-child disconnectedness and parents’ depressive symptoms among N = 216,573 older Europeans across four regions: Northern, Eastern, Southern, and Western Europe. We also test for gender and marital status differences therein. Descriptive analyses show that parent-child disconnectedness is rare in Europe, ranging from 1% among women and men in Southern Europe to 5% among women and 6% among men in Northern Europe. Multivariable pooled OLS show that parent-child disconnectedness is linked to higher depressive symptoms across all regions, with the greatest mental health burden in Southern Europe. These trends are consistent for men and women, but vary across different marital statuses. Our results align with theories suggesting that the psychological toll of disconnectedness is greater in cultures where it is less common, leading to a lack of support and stigma for parents. However, we also find evidence of a more modest burden in other European regions.

